# Laboratory Extractions of Soil Phosphorus Do Not Reflect the Fact That Liming Increases Rye Phosphorus Content and Yield in an Acidic Soil

**DOI:** 10.3390/plants11212871

**Published:** 2022-10-27

**Authors:** Miguel Ángel Olego, Mateo D. Cuesta-Lasso, Fernando Visconti Reluy, Roberto López, Alba López-Losada, Enrique Garzón-Jimeno

**Affiliations:** 1 Research Institute of Vine and Wine, Universidad de León, Avenida de Portugal, 41, 24071 León, Spain; 2 RGA Bioinvestigación S.L., 24071 León, Spain; 3 Departamento de Ecología, Centro de Investigaciones Sobre Desertificación-CIDE (CSIC, UVEG, GVA), Carretera CV-315, km 10.7, 46113 Moncada, Spain; 4 Department of Applied Chemistry and Physics, Faculty of Biology and Environmental Sciences, Universidad de León, Campus de Vegazana, 24071 León, Spain

**Keywords:** biomass, extractant, liming, Mitscherlich equation, phosphorus, soil acidity

## Abstract

In addition to aluminum and other heavy metal toxicities, acidic soils also feature nutrient deficits that are not easily overcome by merely adding the required amounts of mineral fertilizers. One of the most critically scarce nutrients in acidic soils is phosphorus, which reacts with aluminum and iron to form phosphates that keep soil phosphorus availability significantly low. Liming ameliorates acidic soils by increasing pH and decreasing aluminum contents; however, it also increases the amount of calcium, which can react with phosphorus to form low-solubility phosphates. In the present work, three liming materials, namely, dolomitic limestone, limestone and sugar foam, were applied on a Typic Palexerult cropped with rye. The effects of these materials on soil properties, including soil available phosphorus extracted with the Olsen and Bray-1 methods, rye phosphorus content in stems and stem and spike harvested biomasses were monitored for nine years. According to the Olsen extraction, the amount of soil available phosphorus generally decreased following liming, with limestone presenting the lowest values; however, the amount of soil available phosphorus increased according to the Bray-1 extraction, though only to a significant extent with the sugar foam from the third year onward. Regardless, the phosphorus content in rye and the relative biomass yield in both stems and spikes generally increased as a consequence of liming. Since crop uptake and growth are the ultimate tests of soil nutrient availability, the inconsistent stem phosphorus content results following the Olsen and Bray-1 extraction methods suggest a lowered efficiency of both extractants regarding crops in soils rich in both aluminum and calcium ions. This decrease can lead to important interpretation errors in the specific conditions of these limed acidic soils, so other methods should be applied and/or researched to better mimic the crop roots’ phosphorus extraction ability. Consequently, the effects of the liming of acidic soils on phosphorus availability and crop performance in the short and long term will be better understood.

## 1. Introduction

Soil acidity naturally develops (i) on acidic parent rocks; (ii) as a consequence of the leaching of alkali and alkaline earth minerals [1]; (iii) due to the wet and dry atmospheric deposition of acid gases, e.g., $SO_{2}$ and $NO_{X}$, which is usually artificially exacerbated; and (iv) due to plant nutrient uptake and the mineralization of the soil’s organic matter [2]. Although there is evidence that certain organic residues from green and animal manures increase soil pH in croplands [3], it is well-established that the application of acidifying mineral fertilizers appreciably contributes to soil acidification [4], and a low soil pH is a major factor limiting crop fertilization and yields. The reasons for this decrease in plant productivity are an imbalance in both macro- and micronutrients in the soil [5] and the release of appreciable amounts of aluminum (Al) and manganese (Mn) into the soil solution [6], which impairs plant growth due to the elements’ toxicity [5].

Phosphorus is a limiting nutrient for terrestrial biological productivity [7], as its plant uptake is relatively less efficient than most other nutrients due to its significantly low concentrations in soil solutions [8]. The soil solution contains phosphorus in the form of hydrogen tetraoxophosphate (V) ions (both mono- and divalent anions), but monovalent anions are the dominant form in acidic soils. However, in these low-pH soil conditions, Al, iron (Fe) and Mn cations form precipitates with the dissolved monovalent phosphorus anions to form insoluble precipitated phosphates such as variscite $\left(Al{\left(OH\right)}_{2}H_{2}PO_{4}\right)$ and strengite $\left(FePO_{4}\cdot 2H_{2}O\right)$, which keep the phosphorus concentration in the soil solution significantly low; consequently, most soil P becomes almost unavailable to crops [9,10]. In addition to the direct chemical effects indicated so far, there are indirect biological effects that constrain the phosphorus availability to plants in acidic soils. In this regard, plant litter decomposition is important for the recycling of not only carbon but also other nutrients such as phosphorus in soils [11]. Since soil pH exerts a profound effect on soil microbial communities through its effects on nutrient availability [9], biological communities, which are positively correlated with litter decomposition rates [11], tend to become less active and diverse as pH decreases [12]. Thus, since much of the phosphorus taken up by crops is provided via the mineralization of soil organic materials [8], the impacts of soil acidity on microbial diversity and communities indirectly curtail soil phosphorus availability to crops.

The amelioration of soil acidity generally involves liming [13], which comprises the application of ground calcium and/or magnesium carbonate rocks and industrial hydroxides, i.e., lime [14]. In addition to these, several waste resources containing Ca and/or Mg carbonates and hydroxides, including agricultural [15] and industrial by-products [16], are reused for the liming of acidic soils [17]. Liming increases soil solution pH and replaces most of the $Al^{3+}$, $H_{3}O^{+}$ and $Mn^{2+}$ ions with $Ca^{2+}$ and $Mg^{2+}$ ions in both the soil solution and exchange complex, and this ion exchange lessens the toxic effects of the former ions and hence fosters better crop growth and the efficient use of fertilizer nutrients [6]. This technical solution, which addresses the issues of soil acidity, has been comprehensively explained in many publications [2,6,13,18,19].

Though phosphorus uptake and utilization by crops play vital roles in final crop yield and quality, the topic of phosphorus plant nutrition in acidic soils—particularly in limed acidic soils—has not received enough attention in the literature because researchers have thus far focused on the amelioration of soil acidification by primarily evaluating crop productivity, soil pH, aluminum phytotoxicity and the availability of exchangeable cations. Additionally, in studies wherein phosphorus plant nutrition in limed soils was addressed, there have been inconsistent results regarding the effects of liming on soil phosphorus availability [20,21,22].

These inconsistencies could be attributed to variations in the soil phosphorus extraction ability between methods. In this regard, the 0.5 M hydrogen carbonate used in the Olsen extraction is considered the standard for soils from acidic to alkaline pH, whereas the 0.03 M fluoride used in the Bray-1 extraction is only considered the standard for acidic soils [23]. Whatever the case, these methods, regardless of their merits, are proxies intended to recreate the phosphorus extraction ability of the plants’ roots, which is the ultimate benchmark of nutrient availability.

The purpose of this investigation was to explore the long-term (9 years) effects of three liming amendments, namely, limestone, dolomitic limestone and sugar foam, on soil phosphorus availability according to the Olsen and Bray-1 extraction methods, on rye harvested biomass and on phosphorus concentrations in rye stems. The hypothesis that was tested in this work was that ameliorating soil acidity through liming has significant enhancement effects on soil phosphorus availability and, hence, on crop phosphorus uptake and growth, as reflected in biomass build-up.

## 2. Results

### 2.1. Soil Properties before Liming

Although initial soil characterizations regarding texture, soil organic matter (SOM), pH and major cations have already been reported in previous works [24,25], soil phosphorus levels extracted with both sodium hydrogen carbonate (Po) and ammonium fluoride in hydrochloric acid (P1) are presented here for the first time. Furthermore, the calcium (Ca), magnesium (Mg), potassium (K) and aluminum (Al) contents were measured and are expressed as effective exchange capacity percentages in all soil horizons, i.e., CaECEC, MgECEC, KECEC and AlECEC, respectively (Table 1).

Regarding soil phosphorus levels, values between 15 and 25 mg/kg are usually considered correct for low pH, medium-to-sandy-textured soils with low-to-medium phosphorus-demanding crops such as rye [25,26,27,28]. Therefore, as shown in Table 1, soil Po presented low-to-medium agronomic levels in both the Ap1 and Ap2 horizons, though it then sharply decreased to significantly low levels in the underlying AB horizon. On the other hand, P1 only showed low-to-medium agronomic levels in the Ap1 horizon and then showed lower levels in the lower horizons.

The exchangeable Al contents were well over 20% in all horizons. Since the 20% limit is considered the highest Al saturation that most plants can tolerate [19], this characteristic reveals the harsh conditions the study site initially presented for agricultural development. Consequently, significantly low exchangeable Ca and Mg contents at both the Ap1 and Ap2 horizons were also found, though they were somewhat improved at the AB horizon.

### 2.2. Soil Properties after Liming

According to the normality tests carried out on the soil data, Po and SOM could be considered to originate from normally distributed populations and were therefore not transformed. However, the other properties could not be considered the same way and were transformed. The best Box–Cox transformations occurred for P1, CaECEC and AlECEC when using the square root; for MgECEC when using the logarithm; and for pH when using the inverse square root.

According to the ANOVAs, the liming treatment (T) had significant effects on both Po and P1, as well as pH, CaECEC, MgECEC and AlECEC (Table 2). Moreover, there were differences for all soil properties depending on horizon (H) and year (Y). Additionally, the effect of T on every property significantly changed in magnitude or direction with both H and Y, as revealed by the significant interactions among the T, H and Y factors.

**Figure 1:**
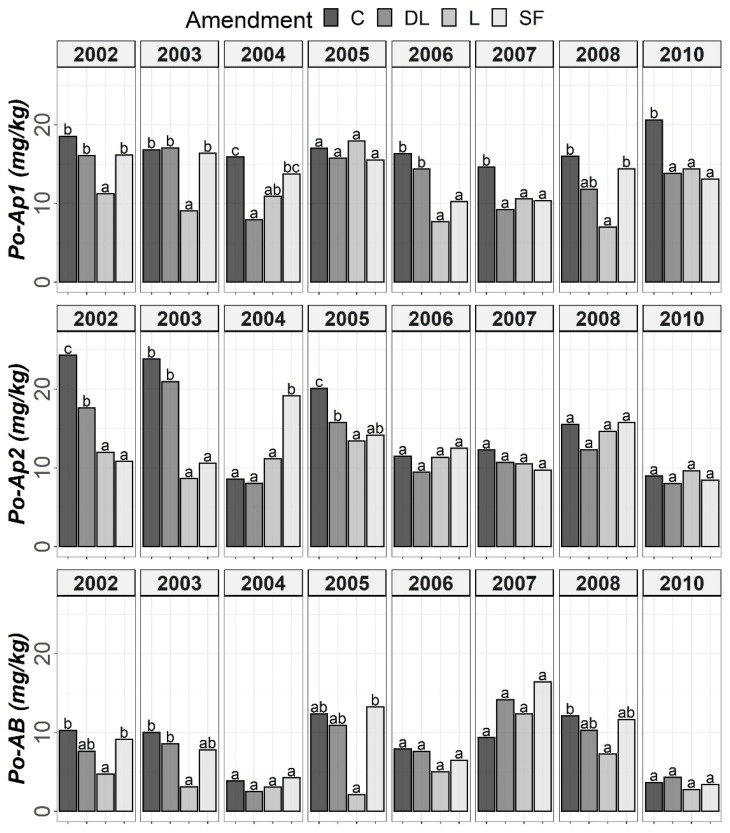
Temporal evolution of Olsen’s phosphorus (Po) in the three horizons (Ap1, Ap2 and AB) studied throughout the soil monitoring period (2002–2010). Bars within each year and horizon followed by different lowercase letters reflect significantly different averages according to Tukey’s honest significance test with Holm–Bonferroni adjustment (*p* = 0.05).

**Figure 2:**
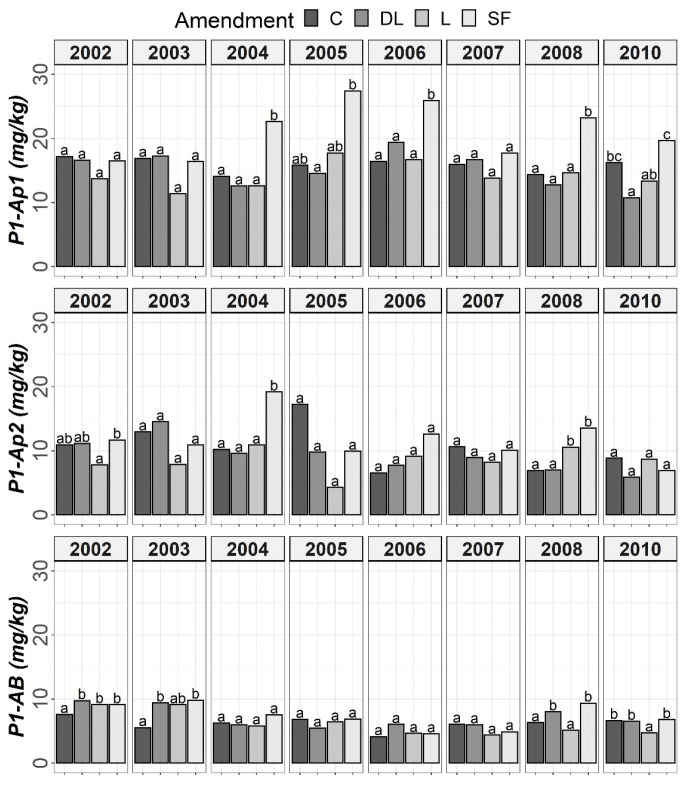
Temporal evolution of Bray-1’s phosphorus (P1) in the three horizons (Ap1, Ap2 and AB) studied throughout the soil monitoring period (2002–2010). Bars within each year and horizon followed by different lowercase letters reflect significantly different averages according to Tukey’s honest significance test with Holm–Bonferroni adjustment (*p* = 0.05).

**Figure 3:**
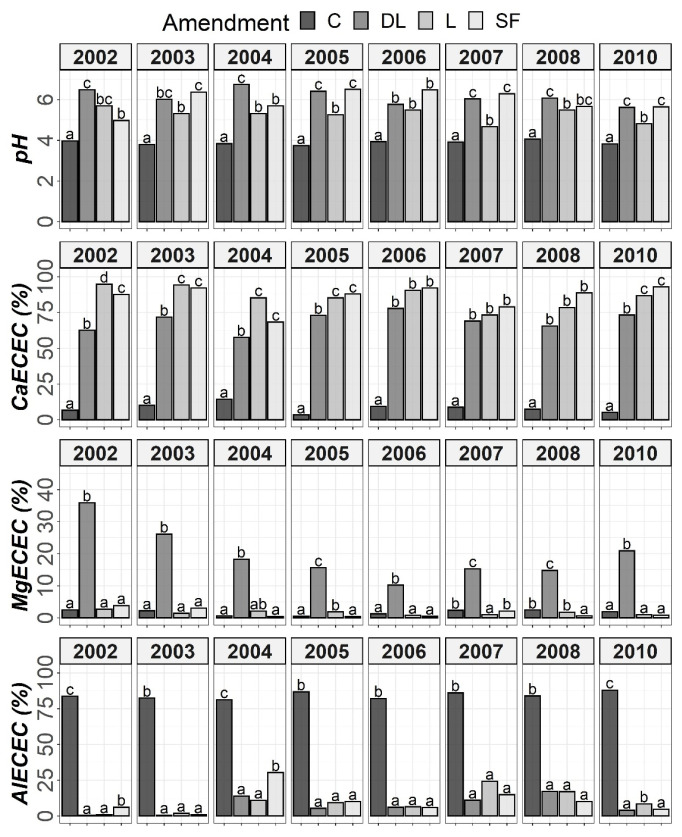
Temporal evolution of pH, CaECEC, MgECEC and AlECEC in the Ap1 horizon throughout the soil monitoring period (2002–2010). Bars within each year followed by different lowercase letters reflect significantly different averages according to Tukey’s honest significance test with Holm–Bonferroni adjustment (*p* = 0.05).

**Figure 4:**
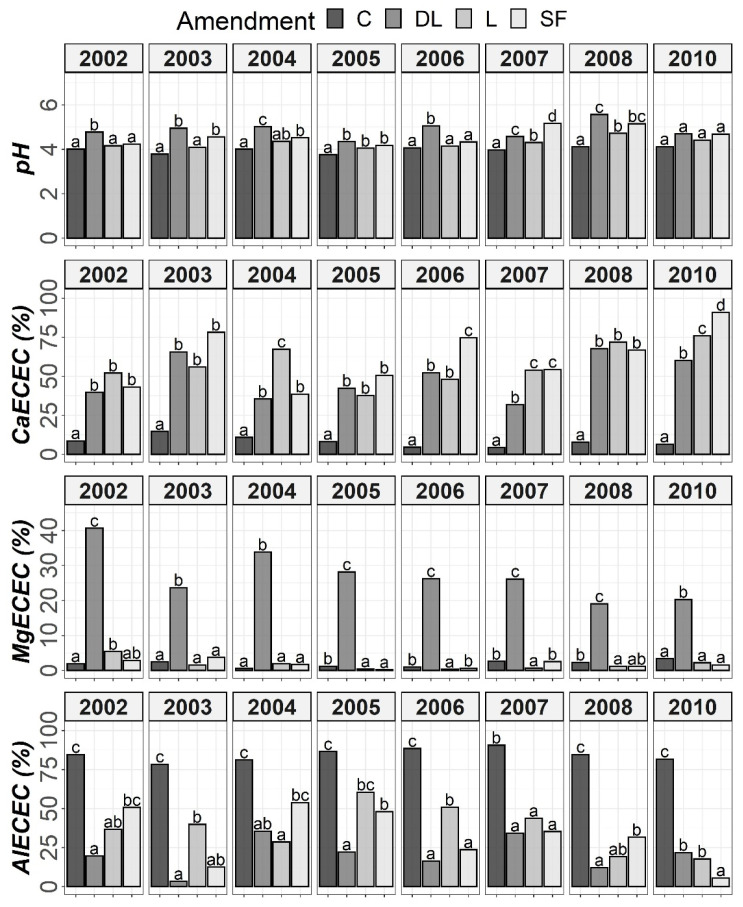
Temporal evolution of pH, CaECEC, MgECEC and AlECEC in the Ap2 horizon throughout the soil monitoring period (2002–2010). Bars within each year followed by different lowercase letters reflect significantly different averages according to Tukey’s honest significance test with Holm–Bonferroni adjustment (*p* = 0.05).

The results of Tukey’s HSD post hoc comparisons based on the ANOVA outcomes among the liming treatments and control subplots throughout the monitoring period are shown in Figure 1, Figure 2, Figure 3 and Figure 4 in terms of both H and Y. Although the temporal evolution of soil pH and Ca, as well as Mg and Al levels in all studied horizons (Ap1, Ap2 and AB) has been documented in previous works [24,25], these temporal evolutions are now considered alongside soil available P to understand the effects of liming materials on soil P availability. In this study, no significant effect of T on SOM was found, so although the significant interaction between T, H, and Y revealed that the effect of T was modulated by both H and Y (Table 2), the corresponding temporal evolution of SOM is not shown.

The soil available Po levels at the Ap1 horizon were generally higher in the control treatments than in the liming treatments from the start of the monitoring. Conversely, at both the Ap2 and AB horizons, no clear differences among the liming treatments and between the liming treatments and the control treatments were found. As a whole, limestone (L) demonstrated the best ability to decrease Po availability among liming materials. This effect may have been linked to the lower soil pH associated with the L treatment in comparison with the DL and SF treatments. On the other hand, from year 2004 onward, a clear trend of higher P1 levels in the SF treatment than in the other treatments was observed at the Ap1 horizon (with these differences being significant relative to the control in years 2004, 2006 and 2008). This trend was also seen for the years 2004 and 2008 at the Ap2 horizon. However, at the AB horizon, no clear differences in Po among the liming treatments or between the liming treatments and the control treatments were found (Figure 1 and Figure 2).

**Figure 5:**
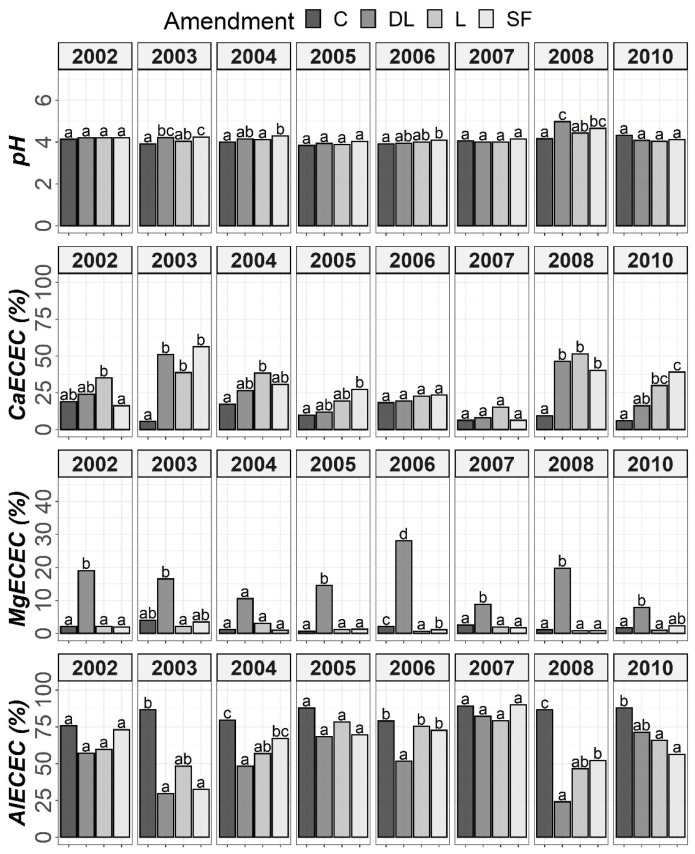
Temporal evolution of pH, CaECEC, MgECEC and AlECEC in the AB horizon throughout the soil monitoring period (2002–2010). Bars within each year followed by different lowercase letters reflect significantly different averages according to Tukey’s honest significance test with Holm–Bonferroni adjustment (*p* = 0.05).

At the Ap1 depth, both the L and SF treatments similarly increased CaECEC levels to over 70% on average from the beginning to the end of the monitoring, whereas the DL treatment showed less CaECEC increases because it noticeably increased MgECEC levels over the same time period (Figure 3). Both the pH and AlECEC levels clearly increased and decreased, respectively, in response to all liming treatments, but pH was generally higher in both the DL and SF treatments than in the L treatment. In the deeper horizons, there were less evident differences in soil available phosphorus levels. The effects of the liming treatments on pH, CaECEC, MgECEC and AlECEC, though milder, still seemed evident at both subsurface horizons, with the L treatment again featuring the lowest soil pH of the liming treatments (Figure 4 and Figure 5). These results reveal that pH affects phosphate adsorption and thus P availability. Additionally, because SOM was not affected by liming and because the mineralization of organic phosphorus provides most of the phosphorus taken up by crops [8], the soil organic fraction did not seem to significantly influence P availability in our research.

According to the mixed ANOVAs, the effect of the limestone (L) treatment decreasing soil Po availability was found to be statistically significant from the first year onward at the Ap1 horizon. On the contrary, no significant trend was found for the DL and SF treatments compared with the control during the first year. In this respect, the first significant difference between the DL and C treatments at this shallow horizon was found in 2004, and that for the SF and C treatments was found in 2007. On the other hand, the effect of the sugar foam (SF) treatment in increasing soil P1 availability was found to be statistically significant from the third year onward at this soil depth. However, no significant trend on P1 was found for both the DL and L treatments compared with the control during the research.

According to data comparison at Ap2 and AB, the L treatment had a more significant decreasing effect on the short-term Po availability than the DL or SF treatments, and then, the differences among liming treatments progressively vanished. In summary, the reduction pattern exhibited by the liming materials on Po availability was the highest in the Ap1 horizon, it was maintained from four to five years, and it was best sustained by the L treatment. On the other hand, as in the Ap1 horizon, the SF treatment demonstrated the most striking effect in comparison to control subplots in the Ap2 horizon (with significant increases in both 2004 and 2008), whereas no significant trend was found for the DL treatment and only a significant increase in 2008 was found for the L treatment. Finally, no significant differences in P1 levels were found between the liming treatments in the AB horizon.

### 2.3. Olsen vs. Bray-1 Soil Phosphorus Extractions

**Figure 6:**
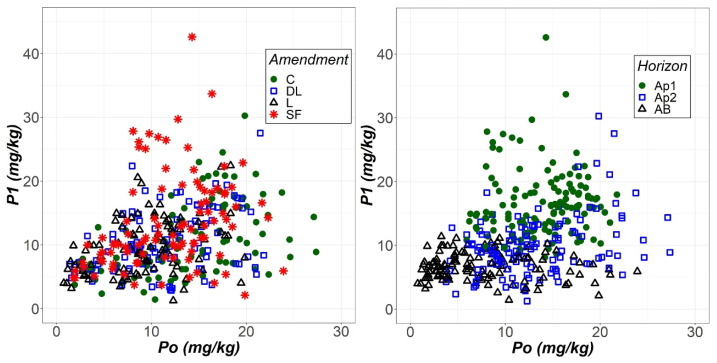
Scatterplots of the phosphorus extracted with the Olsen (Po) and Bray-1 (P1) methods as a function of both liming treatment (control (C), dolomitic limestone (DL), limestone (L) and sugar foam (SF)) and soil horizon (Ap1, Ap2 and AB).

The soil phosphorus levels extracted with the Olsen (Po) and Bray (P1) methods were compared through scatter graphs (Figure 6). The most compelling aspect of these graphs is that P1 seemed to be more sensitive than Po to the liming effect of the SF treatment but less likely than Po to show an increase in soil phosphorus levels as soil depth increased.

### 2.4. Rye Stem Phosphorus Content and Biomass after Liming

Similar to soil available phosphorus, the evolution of the rye stem phosphorus content (P-Stem) and the harvested stem and spike biomasses (Stem and Spike, respectively) throughout the monitoring period was studied. The temporal evolution of the rye stem, calcium and magnesium contents were extensively documented in previous works [24,25], so they are not dealt with here.

Before conducting the ANOVAs, data distributions were explored. Biomass data, i.e., Spike and Stem data, did not require any transformation, but P-Stem data were transformed using the logarithm. Next, according to the two-way ANOVAs, there was a significant effect of T on P-Stem, as well as on Spike and Stem. Furthermore, the effect of T was modulated by Y, as revealed by their significant interaction (Table 3).

**Figure 7:**
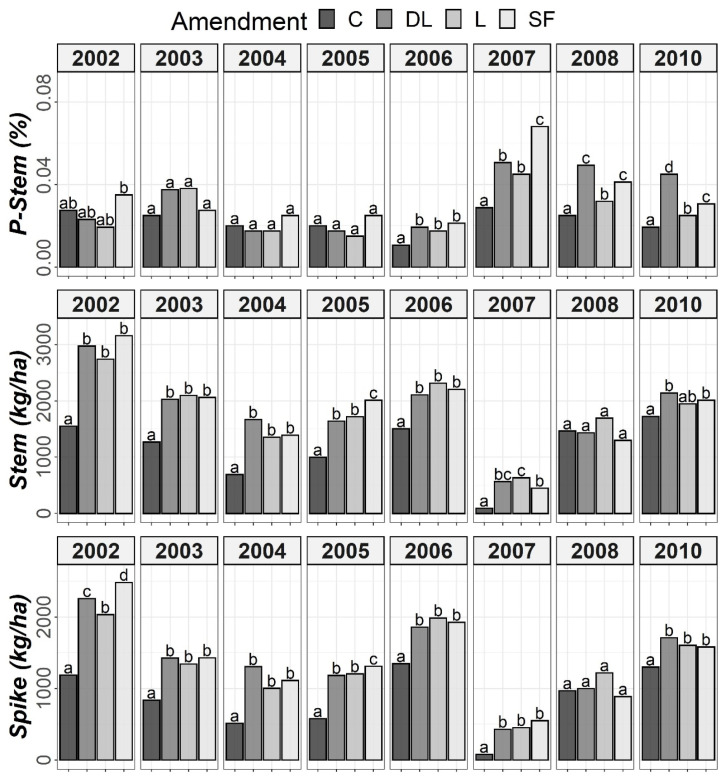
Temporal evolution of phosphorus levels in stems (P-Stem) and both Stem and Spike biomass throughout the research period (2002–2010). Bars within each year followed by different lowercase letters reflect significantly different averages according to Tukey’s honest significance test with Holm–Bonferroni adjustment (*p* = 0.05).

According to the ANOVA outcomes, the rye data, similarly to the soil data, had to be split for Tukey’s post hoc tests. As shown in Figure 7, a marked increase from the sixth year (2007) onward was found for P-Stem in the limed subplots compared to the controls. For the five first years (2002–2006), no marked differences in P-Stem levels were observed between the control and liming treatments, except for 2003 in both the DL and L treatments. Specifically, the SF treatment showed the highest P-Stem values in 2007, whereas the DL treatment more consistently showed higher values than those of the C subplots during the years 2007–2010. Additionally, both Spike and Stem were consistently higher in the DL, L and SF treatments compared to the C treatment, especially in 2007, which was characterized by both a low rainfall and a low average annual temperature. The highest peak in P-Stem in all liming subplots occurred that year, which was a remarkable observation.

Seen as the differences in biomass from year to year are strongly influenced by weather and related variables, the trends in relative yield (i.e., the yield rescaled to the maximum value for each year) in both Spike and Stem were evaluated. However, the effect of T on both relative yields was also shown to be modulated by Y. Moreover, the same post hoc results were achieved (graphs not shown).

### 2.5. Relative Stem and Spike Yields against Phosphorus in Soil and Stem

Although the same statistical results were obtained for both absolute and relative yields, the relationships between both the Stem and Spike relative yields and the total soil available phosphorus (as determined with the Olsen or Bray-1 methods) were assessed. In this regard, the phosphorus contents of the upper two horizons were added together (∑P1), which is consistent with the reported 25 cm maximum depth for the rye rooting system [29]. In these calculations, an estimate of 1.58 g/cm^3^ for the bulk density of both the Ap1 and Ap2 horizons was used on the basis of the textural class according to the USDA [30]. Additionally, the relationships between the Stem and Spike relative yields with P-Stem were evaluated.

**Figure 8:**
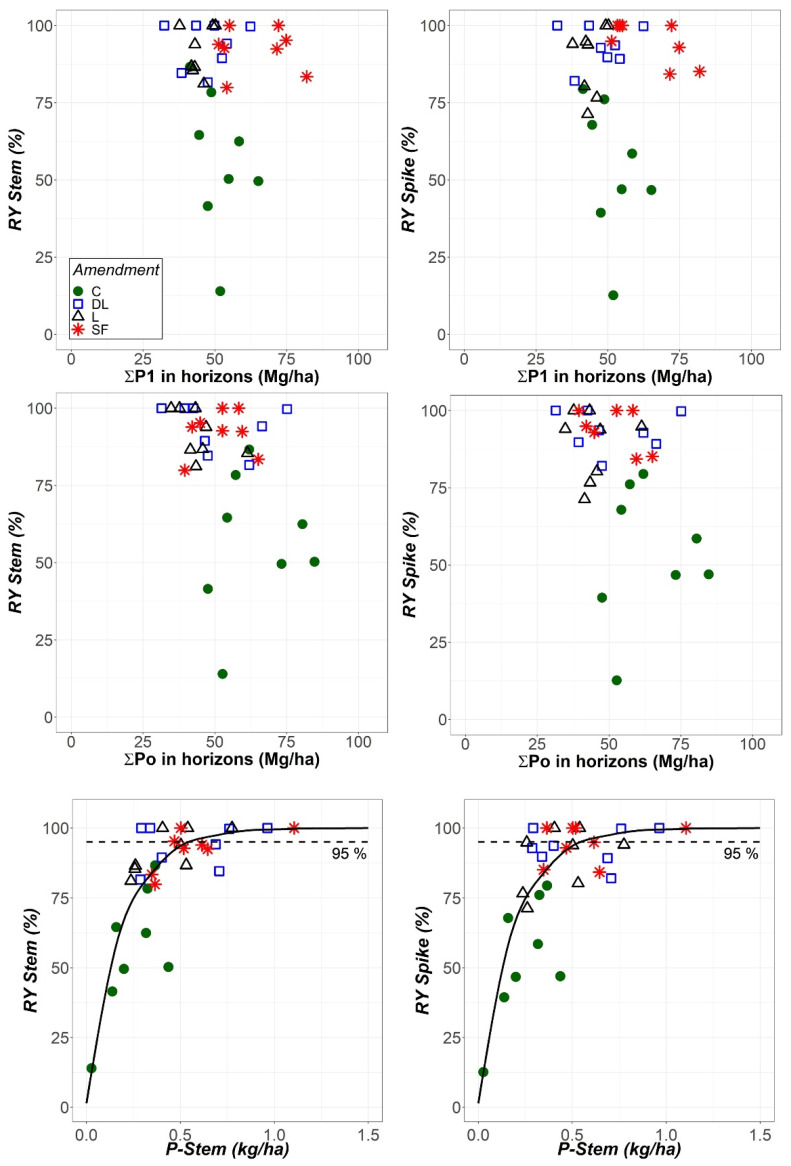
Relationship between the relative yields of stems (RY Stem) and spikes (RY Spike) and the sum of total soil available P in the Ap1 and Ap2 horizons as determined by using the Bray-1 and Olsen methods (P1 and Po, respectively), as well as between both RY Stem and RY Spike and the P content in stems (P-Stem) at the harvest stage. All results are expressed as a function of the liming treatment (control (C), dolomitic limestone (DL), limestone (L) and sugar foam (SF)) as indicated in in upper left panel. Mitscherlich curves passing through the origin were fit to the data of RY against P-Stem.

As shown in Figure 8, there was no apparent relationship between both relative Stem and Spike yields and total soil available phosphorus, as determined with either the Olsen or Bray-1 methods. Therefore, in contrast to what was previously considered, a higher amount of soil available phosphorus does not imply an increase in relative rye biomass in either spikes or stems. Conversely, a higher P-Stem content implies a higher relative yield that gradually decreases up to a limit. These results are in accordance with the Mitscherlich law of diminishing returns. Therefore, the Mitscherlich curves forced to pass through the origin were fit to the data. For the observed curves at a 95% relative spike yield, mean ± 95% confidence interval values of 0.57 ± 0.12 kg/ha and 340 ± 90 mg/kg DW for the P-Stem content were obtained.

## 3. Discussion

The improvement of the soil’s chemical attributes as a consequence of liming observed in this research has been extensively reported in previous works [24,25]. However, the main aim of this study was to consider the effects of liming on phosphorus availability in acidic soils. In the current study, soil available Po decreased following liming, especially with L, which also demonstrated the lowest pH among the liming treatments. On the contrary, from halfway through the duration of the research onward, the soil available P1 showed an increasing trend when the liming material was SF; for this research period, this liming material also showed the highest CaECEC levels. In previous studies, a decreasing effect of liming on soil available phosphorus in acidic soils was shown by Park and Ro [20] and Qaswar et al. [21], but the opposite was shown by Mkhonza et al. [22].

Park and Ro [20] showed that high doses of liming materials can negatively impact soil available phosphorus due to inorganic phosphorus’ fixation with Ca. In this research, the lime requirement when using Cochrane’s formula yielded a value of about 6.4 Mg CCE/ha, a higher lime rate than those typically used by growers in the area, which are usually below 1.0 Mg CCE/ha. Due to the fact that the lime rate raised the soil pH by more than one unit in the Ap1 horizon, it could be considered sufficiently high. A possible explanation for the Po results may be that although liming improves the conditions for plant growth in acidic soils by reducing the concentration of soluble phytotoxic ions such as the aluminum ones, which otherwise react with phosphorus to form low-solubility aluminum phosphates, it also increases phosphate fixation due to the formation of relatively insoluble calcium phosphates when pH values above neutrality are attained [31].

In this study, pH values above neutrality were not achieved. Therefore, as suggested by Simonsson et al. [32], it would be reasonable to hypothesize that the phosphorus in combination with the 8/15/15 background complex fertilizer may have been primarily associated with Al through sorption by surface aluminol groups (Al-OH) rather than through mineral precipitation. In this regard, calcium ions could have acted as cation bridges for binding more phosphate on the soil surface [33]. However, it is surprising that although the observed delayed increase in CaECEC levels in the SF subplots from halfway through the duration of the research onwards could be attributed to the low fineness of this liming material, they were coincident in time with higher P1 levels. A possible explanation for this may be a soil amelioration in terms of pH neutralization, microbial biomass and enzymatic activity supported by the SF treatment as a sustainable technique to induce soil physical, chemical and biological fertility [34]. Moreover, the low fineness of SF could be a possible explanation for higher values of P1 in comparison with Po in certain SF-limed subplots.

Contrary to the results obtained in this work, Mkhonza et al. [22] observed increasing soil phosphorus availability extracted with ammonium bicarbonate in their acidic soils as a consequence of liming, and they explained this effect by suggesting that both Al and Fe oxides became more negatively charged with increasing pH, thus contributing to the desorption of phosphate ions from mineral surfaces and increases in the soil available phosphorus. Liming may also increase soil phosphorus availability by stimulating the mineralization of soil organic phosphorus [31]. However, in the presented study, none of the liming materials had a significant effect on the soil organic matter (SOM) levels throughout the soil profile (Table 2 and [24]); therefore, they could not have contributed to increases in soil phosphorus availability in the conditions of this field experiment.

Additionally, the high fineness of L, particularly in comparison to SF, may have contributed to the differences of pH and thus the available Po of this treatment with respect to the others. Along with CCE, fineness defines the material quality of a liming agent [19] because it controls its dissolution rate. In this work, fineness was only qualitatively assessed, but it could be conceivably hypothesized that the different effects of L on pH and Po could be attributed to a higher degree of fineness. These results contrast those regarding CCE, whose value for the L treatment was between those of the DL and SF treatments, so it does not seem to be able to explain the observed differences among the liming treatments.

The diminishing magnitude of the differences in soil available phosphorus between liming treatments and control subplots as soil depth increased may be partly explained by two phenomena. First, it may have been caused by the slow downward movement of $Ca^{2+}$ and $Mg^{2+}$ after the exchangeable sites in the topsoil were saturated [35]. Second, it may have been caused by the higher particle size of the Al and Fe oxides in both sandy loam Ap1 and Ap2 horizons than in the sandy clay loam AB horizon, thus indicating more labile phosphorus pools and hence a higher potential for the enhancement of phosphorus extraction with improved soil conditions in shallower horizons [36].

Two sources of uncertainty must be highlighted here. The first one is that the top 5 cm of soil was discarded during the soil samplings in both the control and liming treatments. Therefore, in the decreasing trend of available phosphorus through soil depth, a possible bias could have been observed due to a faster rate of lime dissolution in the soil layer that was discarded. The second source of uncertainty was that the slow reactions between phosphate and soil (caused by several factors such as soil temperature) could have affected the effectiveness of the phosphate application during the first two years, which could have affected subsequent crop seasons [37]. In this regard, it is possible that the soil phosphorus results obtained in certain liming treatments and years may be biased due to the continued slow reactions of phosphorus with the soil over time. In future investigations, some measure of the ability of soil to retain soil phosphorus would be useful to assist with the interpretation of results.

Regarding the soil available phosphorus levels before liming, only somewhat low estimates could be made since no specific information for rye could be found [38]. Additionally, the soil phosphorus availability according to the extracted Po and P1 levels did not increase as a consequence of liming, so the phosphorus availability could be regarded as still being low after the treatments. However, the relative yield in terms of both stems and spikes did increase following liming, and part of this increase could be attributed to the amelioration of rye phosphorus uptake according to the Mitscherlich law fit of the relative yield data to the phosphorus stem content. Furthermore, the maximum relative yield was never attained in the control treatment in any year, only in the different liming treatments (Figure 8 and related text). Conversely, the lack of relationship between both the spike and stem yields with both Po and P1 indicates that these soil extractions could not reflect that fact (Figure 8). Therefore, regardless of the mechanism underlying the observed trends of both available Po and P1 in the limed subplots, liming actually fostered phosphorus build-up in rye with consequences on yield increase. The increases in both CaECEC and MgECEC, as well as the concomitant decreasing in AlECEC in liming subplots from the first year onward, could explain the increase in Spike and Stem, as also shown in previous studies [24,25]. Additionally, as soil pH increases and thus approaches neutrality, a shift in the balance between various phosphorus sorption mechanisms may occur [39]. Therefore, phosphorus root uptake can increase while Po and P1 remain unaffected or even decrease. The results in this investigation accordingly suggest that the improvement of the soil conditions for plant growth could have worked, at least in part, through the amelioration of phosphorus rye nutrition. This improvement of phosphorus nutrition occurred not only due to the enhancement of the soil conditions with liming, which increased Ca and Mg nutrient contents and lowered Al toxicity, but also due to likely changes in the phosphorus sorption status in the soil. These changes enabled the rye roots to uptake more phosphorus despite the availability that was observed on the basis of the laboratory extractions.

The issue of available phosphorus levels when liming acidic soils is intriguing [20,33]. However, the ultimate benchmarks for phosphorus and other nutrient availabilities in soils are crop uptake and development, not a laboratory proxy such as the Olsen and Bray-1 extractions used for phosphorus. In this study, the laboratory-observed lower availability of Po in limed subplots, especially when limestone was used, was not detected in the rye phosphorus uptake and biomass build-up tests. Therefore, was there a real lower phosphorus availability in those limed plots? The Olsen soil phosphorus extraction method has been shown to depend on soil properties such as pH and CCE more than other methods such as Mehlich 3 [40,41]. These results may be explained by the fact that the soil phosphorus uptake of crops also depends on the phosphorus buffering capacity of soil, and methods such as the anion exchange resin phosphorus extraction also depend on this property more than both the Olsen and Bray-1 methods [42,43]. Therefore, other laboratory proxies for soil available phosphorus, specifically those less dependent on soil properties such as pH and CCE and more dependent on the soil’s phosphorus buffering capacity, should be applied and further investigated. This is particularly important for soils derived from limed acidic soils in which phosphorus must be extracted from Al and Ca phosphates in the forms of precipitated minerals and surface complexes.

## 4. Materials and Methods

### 4.1. Study Site

The soil under study corresponded to an acidic Typic Palexerult (USDA, 2010), located in the village of Camposagrado (municipality of Rioseco de Tapia, León, Spain). This kind of soil, which is well-represented in the northern regions of the Iberian Peninsula, mostly occurs in old “raña” surfaces. These are sedimentary formations comprising quartzite pebbles with a clay matrix that date back to the middle–upper Pliocene when they developed at the piedmont of quartzite ranges. Therefore, the “raña” soils have inherited their acidity from their parent materials. Though fairly flat, these soils are subjected to the major agronomic shortcoming of a natural argillic horizon with a low pH, a high content of exchangeable aluminum and a low contents of essential elements [44].

The presented research evaluated a *Secale cereale* L. crop (long-cycle rye variety named “Ordalie”) over a period of nine cropping years (2002–2010). The main characteristics of the study site (including the Universal Transverse Mercator coordinates, altitude and bioclimatic characteristics), the amount of seeds and the local fertilization scheme were described in a previous work [24].

### 4.2. Characterization of the Liming Materials and Its Doses

The chemical compositions of the three liming materials used in this study, as well as their calcium carbonate equivalent (CCE) as an expression of the acid neutralizing capacity with reference to that of pure calcium carbonate, were described in our previous works [24,25]. In addition to the composition data therein, it is worth indicating here that the liming materials’ fineness qualitatively increased in the following order from low to high: SF < DL ≈ L. Regarding composition, although limestone (L) showed the highest CaO content, dolomitic limestone (DL) exhibited a higher CCE due to the lighter molar mass of magnesium relative to calcium. In the sugar foam (SF), CaO is mainly present in the form of slaked lime ($Ca{\left(OH\right)}_{2}$) rather than carbonate ($CaCO_{3}$) [45]. Therefore, to add the same CaO content of the liming materials in reference to the CCE values, the lime requirement (LR) was calcium-based and calculated using the known Cochrane’s formula [18], taking the exchangeable contents of Ca, Mg and Al into account and targeting an aluminum percentage saturation of below 20%. Details on Cochrane’s formula were documented in previous works [24,25]. In Cochrane’s formula, the Al, Ca and Mg contents of the Ap1 and Ap2 horizons, as well as a crop factor (f) of 6 (on the basis of previously Büchner funnel tests), were used to estimate the LR. Liming materials were manually incorporated by one-pass rotovator tillage at a depth of about 20 cm in September 2001. In the same way as the liming subplots, the one-pass rotovator tillage was carried out in control subplots.

The following local fertilization scheme was used: 150 kg/ha of background fertilizer in the form of an 8/15/15 complex (12.0 kg/ha of N, 22.5 kg/ha of $P_{2}O_{5}$ and 22.5 kg/ha of $K_{2}O$) in September the first two years (2001 and 2002), with a top dressing application of 38.0 kg/ha of N as 33.5% ammonium nitrate in April every year. These fertilizers were spread by hand onto the entire surface of the subplots, but they were not incorporated down into the soil.

### 4.3. Experimental Design

Both the experimental design and statistical analyses were similar to those described in a previous work [24]. In this experimental design, the factors were the liming treatment (T) with four levels (control (C), limestone (L), dolomitic limestone (DL) and sugar foam (SF)), the soil horizon (H) with three levels (Ap1, Ap2 and AB) and the sampling year (Y) with nine levels (2002–2008 and 2010). It is worth noting that there was a gap in 2009, but all the soil and biomass parameters were monitored in the remaining years.

### 4.4. Soil and Biomass Sampling and Analyses

Sampling dates, as well as both soil and biomass analyses, were described in detail in a previous work [24]. Briefly, for soil samples, two auger cores were taken, discarded the top 5 cm per subplot in September each year and then thoroughly blended together to obtain a representative composite sample. This soil sampling was aimed at obtaining an accurate picture of the phenomenon being studied, as well as at accounting for within-plot variability.

The soil available phosphorus was determined via ultraviolet–visible spectroscopy after extraction with 0.5 M sodium hydrogen carbonate at pH 8.5 and with 0.03 N ammonium fluoride in 0.025 N hydrochloric acid following the Olsen and Bray-1 methods, respectively [46]. The phosphorus concentration in the rye stems was determined with inductively coupled plasma atomic emission spectroscopy (ICP-AES) after wet digestion with an acidic mixture of perchloric, sulfuric and nitric acid at 420 °C for 20 min [47]. It is worth noting that at harvest, the stem phosphorus content in cereals is correlated with the spike phosphorus content (e.g., [48]). Therefore, the former could be conceivably used as a proxy for the assessment of the plant phosphorus nutritional status. Finally, the results of all biomass analyses are reported on a dry weight basis.

### 4.5. Statistical Analyses

The statistical analyses of the soil and dry biomass parameters were performed by means of several mixed-effects three-way ANOVA models. For soil data, T and H were the fixed effect factors and Y was the random effect factor. This mixed design was carried out to determine whether there were significant differences among liming treatments and among soil horizons and years, as revealed by the soil chemical and biomass data, particularly those regarding phosphorus. Additionally, the interactions between the three factors were also assessed. For biomass data, T was the fixed effect factor and Y was the random effect factor, so only the interaction between T and Y was assessed. In cases when there were significant interactions among factors, any main effect could not be directly assessed on the basis of either the three or two-way ANOVA results because the interaction superseded it. Therefore, the effects of the liming treatments were then independently studied on the basis of the maximum-likelihood ratios of factor interactions (Table 2 and Table 3). Finally, with the aim of evaluating where significant differences between T averages originated from, further comparisons among liming treatments were carried out via Tukey’s honest significant different test.

Although the mixed ANOVA is robust in terms of the error rate associated with violations of both assumptions of normality and homogeneity of variance (homoscedasticity) when sample sizes are equal [49], for the sake of data analysis convenience, the Box–Cox power functions were applied to the data to compute the optimal transformations of the non-normal dependent variables into normal variables. The software R, in its version 4.1.3 (Vienna, Austria) [50] was used to perform all statistical analyses.

Additionally, the averages and standard deviations of both soil and biomass data are shown in the Supplementary Materials (Tables S1 and S2).

## 5. Conclusions

In this research, it was hypothesized that the amelioration of soil acidity through liming has significant enhancement effects on soil phosphorus availability and that this could be revealed with the widely used Olsen and Bray-1 laboratory methods and with crop phosphorus uptake and growth, as reflected by biomass build-up at harvest. However, none of the laboratory method results agreed with the amelioration results in terms of phosphorus content in the rye stem and the relative yield increase. The results of this research support the idea that regardless of the levels shown by either the Olsen or Bray-1 phosphorus extraction methods, both rye biomass production and stem content increase as a consequence of liming. Thus, liming can adequately aid phosphorus rye nutrition under the highly demanding acidic conditions of the Typic Palexerult soil featured in this research.

This apparent contradiction between both extraction methods and the crop phosphorus content and performance suggests the likely limitations of both laboratory methods to mimic the crop roots’ extraction ability in limed acidic soils. This specific soil may have presented a challenging situation for both extractants because it is both acidic and high in calcium. Therefore, it still presents a lower-than-neutral pH with not only relevant exchangeable and soluble aluminum but also highly exchangeable and soluble calcium. Therefore, the extractants must extract P from both Al and Ca phosphates in the form of both precipitated minerals and adsorbed complexes.

The definite benchmarks for phosphorus and other nutrient availability are crop uptake, development and growth. In order to gain insight into soil acidity management by means of liming, further investigation into crop proxies for soil phosphorus extraction should be carried out in these particularly challenging soils. Otherwise, interpretation errors could lead to phosphorus over-fertilization or soil under-liming. By using methods that better mimic the crop roots’ soil phosphorus extraction ability, the short- and long-term effects of lime application on soil phosphorus availability, as well as on uptake, translocation and utilization by crops, will be better understood.

## Figures and Tables

**Table 1:** Baseline soil horizon characteristics before liming for Ap1 (0–12 cm), Ap2 (12–25 cm) and AB (25–35 cm) horizons (*n* = 3).

Soil Horizon	Textural Class ^a^	pH	SOM ^b^	Po ^c^	P1 ^c^	CaECEC ^d^	MgECEC ^d^	KECEC ^d^	AlECEC ^d^
Ap1	Sandy loam	3.99	2.27	16.5	17.1	6.45	3.87	5.81	83.9
Ap2	Sandy loam	4.03	2.06	16.5	10.9	8.55	2.63	3.95	84.9
AB	Sandy clay loam	4.13	1.03	4.40	7.59	17.5	2.34	2.34	77.8

^a^ Textural class from the United States Department of Agriculture Textural Classification System; ^b^ SOM in %; ^c^ Po and P1 in mg/kg; ^d^ CaECEC, MgECEC, KECEC and AlECEC in %.

**Table 2:** Analysis of variance performed on the soil parameters (pH, CaECEC, MgECEC, AlECEC, Po, P1 and SOM). The variability in the soil parameters was evaluated using the hierarchical multilevel model (maximum-likelihood (ML) ratio). T: liming treatment; H: horizon; Y: year. The results were significant at * *p* < 0.05, ** *p* < 0.01 and *** *p* < 0.001.

Soil Parameter	ML Ratio (T)	ML Ratio (H)	ML Ratio (Y)	ML Ratio (T × H)	ML Ratio (T × H × Y)
pH	130 (***)	239 (***)	18.9 (**)	228 (***)	203 (***)
CaECEC	285 (***)	197 (***)	164 (***)	141 (***)	369 (***)
MgECEC	377 (***)	3.97 (0.14)	25.8 (***)	14.5 (*)	173 (***)
AlECEC	195 (***)	250 (***)	24.9 (***)	157 (***)	237 (***)
Po	37.9 (***)	130 (***)	35.3 (***)	10.3 (0.11)	311 (***)
P1	20.3 (***)	302 (***)	31.1 (**)	16.2 (*)	196 (***)
SOM	2.10 (0.55)	520 (***)	24.1 (**)	9.08 (0.17)	138 (***)

**Table 3:** Analysis of variance performed on stem and spike biomasses (Stem and Spike, respectively) and P content in stems (P-Stem) at the harvest stage. The variability in the biomass parameters was evaluated using the hierarchical multilevel model (maximum-likelihood (ML) ratio). T: liming treatment; Y: year. The results were significant at * *p* < 0.05, ** *p* < 0.01 and *** *p* < 0.001.

Biomass Parameter	ML Ratio (T)	ML Ratio (Y)	ML Ratio (T × Y)
Stem	72.7 (***)	40.3 (***)	66.9 (***)
Spike	98.1 (***)	43.3 (***)	86.9 (***)
P-Stem	33.7 (***)	31.6 (***)	59.5 (***)

## Data Availability

Not applicable.

## Supplementary Materials

The following are available online at https://www.mdpi.com/article/10.3390/plants11212871/s1. Table S1: Averages and standard deviations (SD) of soil properties pH, CaECEC, MgECEC and AlECEC (effective cation exchange capacity of calcium, magnesium and aluminum, respectively, in cmol (+)/kg), Po and P1 (in mg/kg) and SOM (in %) during 2002–2010. Y: Year of sampling; H: soil horizon of sampling (Ap1 horizon: 0–12 cm; Ap2 horizon: 12–25 cm; AB horizon: 25–35 cm); T: liming treatment (C: control; DL: dolomitic limestone; L: limestone; SF: sugar foam). Table S2: Averages and standard deviations (SDs) of biomass (Spike: spike rye biomass; Stem: stem rye biomass (all of them in kg/ha)) and stem phosphorus levels (P-Stem (%)) during 2002–2010. Y: Year of sampling; T: liming treatment (C: control; DL: dolomitic limestone; L: limestone; SF: sugar foam).
