# Supplementary material for: Laboratory Extractions of Soil Phosphorus Do Not Reflect the Fact That Liming Increases Rye Phosphorus Content and Yield in an Acidic Soil
Source: Plants (Basel). 2022 Oct 27;11(21):2871. doi: 10.3390/plants11212871 (PMC9653810; doi:10.3390/plants11212871)
Supplement: Supplementary file 1 [file plants-11-02871-s001.zip › plants-1839756-supplementary.pdf]

plants

Article – Supplementary materials

Laboratory Extractions of Soil Phosphorus Do Not Reflect the Fact That Liming Increases Rye Phosphorus Content and Yield in an Acidic Soil

Miguel Ángel Olego 1,* , Mateo D. Cuesta-Lasso 2, Fernando Visconti Reluy 3, Roberto López 4, Alba López-Losada 1 and Enrique Garzón-Jimeno 1,2

1 Research Institute of Vine and Wine, Universidad de León, Avenida de Portugal, 41, 24071 León, Spain
2 RGA Bioinvestigación S.L., 24071 León, Spain
3 Departamento de Ecología, Centro de Investigaciones sobre Desertificación-CIDE (CSIC, UVEG, GVA), Carretera CV-315, km 10.7, 46113 Moncada, Spain
4 Department of Applied Chemistry and Physics, Faculty of Biology and Environmental Sciences, Universidad de León, Campus de Vegazana, 24071 León, Spain
* Correspondence: molem@unileon.es

SUPPLEMENTARY MATERIALS

Table S1. Averages and standard deviations (SD) of soil properties pH, CaECEC, MgECEC and AlECEC (effective cation exchange capacity of calcium, magnesium and aluminium respectively, in cmol (+)/kg), P (in mg/kg) and SOM (in %) during 2002-2010. Y: Year of sampling; H: Soil horizon of sampling (Ap1 horizon: 0-12 cm; Ap2 horizon: 12-25 cm; AB horizon: 25-35 cm); T: Liming treatment (C: control; DL: dolomitic limestone; L: limestone; SF: sugar foam).

Y	H	T	pH		CaECEC		MgECEC		AlECEC		P		SOM	
			Average	SD	Average	SD	Average	SD	Average	SD	Average	SD	Average	SD
2002	Ap1	C	3.97	0.01	6.70	0.81	2.51	1.34	83.8	4.94	18.5	1.18	2.27	0.53
2002	Ap1	DL	6.49	0.60	62.6	4.54	35.8	4.26	0.34	0.12	16.1	2.26	2.35	0.61
2002	Ap1	L	5.70	0.86	94.9	1.27	2.75	0.76	0.88	0.52	11.2	1.89	2.26	0.48
2002	Ap1	SF	4.98	0.51	87.7	4.14	3.83	1.61	6.16	3.94	16.1	0.78	2.16	0.23
2002	Ap2	C	4.01	0.02	8.73	4.83	1.91	0.75	84.7	6.07	24.3	3.42	2.06	0.54
2002	Ap2	DL	4.78	0.57	39.7	4.48	40.6	18.8	19.6	26.4	17.6	2.29	1.69	0.41
2002	Ap2	L	4.16	0.06	52.1	6.44	5.44	0.19	36.8	4.53	12.0	5.23	2.44	0.53
2002	Ap2	SF	4.24	0.09	43.2	28.6	2.88	1.39	50.7	29.2	10.8	2.45	2.20	0.18
2002	AB	C	4.13	0.04	19.0	3.83	2.08	1.14	75.7	3.70	10.3	1.66	1.03	0.54
2002	AB	DL	4.19	0.14	24.1	9.09	19.0	2.89	57.2	14.0	7.63	1.50	0.72	0.10
2002	AB	L	4.21	0.15	35.0	16.0	2.18	0.53	59.8	15.9	4.74	2.85	0.90	0.45
2002	AB	SF	4.20	0.23	16.2	6.12	1.98	0.90	73.2	16.0	9.13	3.78	1.04	0.13
2003	Ap1	C	3.79	0.07	10.1	2.84	2.20	1.61	82.3	1.52	16.8	1.23	1.92	0.32
2003	Ap1	DL	6.02	0.60	71.7	3.66	26.1	4.72	0.65	0.44	17.1	2.88	1.81	0.18
2003	Ap1	L	5.31	0.15	94.3	4.04	1.48	0.40	1.95	2.13	9.05	1.26	2.13	0.26
2003	Ap1	SF	6.37	0.99	92.2	6.94	3.06	2.46	0.89	0.17	16.4	2.34	1.70	0.23
2003	Ap2	C	3.79	0.04	14.6	3.98	2.46	0.44	78.4	3.74	23.8	1.09	1.98	0.28
2003	Ap2	DL	4.95	0.57	65.6	19.1	23.7	15.7	3.37	3.30	21.0	0.98	1.74	0.16
2003	Ap2	L	4.09	0.17	56.1	33.1	1.55	0.83	39.9	33.5	8.63	1.94	1.92	0.18
2003	Ap2	SF	4.56	0.07	78.3	5.50	3.73	2.50	12.6	4.12	10.6	3.59	1.38	0.26
2003	AB	C	3.91	0.03	5.55	5.03	4.03	3.14	86.6	5.24	10.0	0.62	0.90	0.43
2003	AB	DL	4.22	0.21	50.9	14.6	16.5	13.7	29.7	22.8	8.55	3.70	0.97	0.42
2003	AB	L	4.04	0.08	38.8	7.39	2.18	0.61	48.3	20.6	3.12	1.25	0.80	0.05
2003	AB	SF	4.25	0.10	56.3	6.62	3.52	2.16	32.6	12.9	7.81	4.80	0.94	0.28
2004	Ap1	C	3.84	0.05	14.4	2.53	0.58	0.31	81.3	5.72	15.9	3.14	2.40	0.33
2004	Ap1	DL	6.74	0.52	57.6	4.21	18.3	14.4	14.0	5.86	7.92	0.72	2.30	0.11
2004	Ap1	L	5.32	0.65	85.4	3.59	2.15	1.23	11.0	3.11	10.9	1.82	2.01	0.15
2004	Ap1	SF	5.70	0.90	68.3	9.92	0.45	0.30	30.3	10.6	13.7	2.15	2.42	0.38
2004	Ap2	C	4.01	0.25	10.8	7.05	0.60	0.50	81.2	13.6	8.58	4.68	2.20	0.94
2004	Ap2	DL	5.01	0.27	35.4	5.89	33.8	10.0	35.6	16.9	8.05	3.60	1.83	0.52
2004	Ap2	L	4.36	0.24	67.4	19.3	2.00	1.33	28.6	20.2	11.1	3.45	2.34	0.11
2004	Ap2	SF	4.52	0.30	38.6	5.52	1.68	1.63	53.9	14.4	19.1	1.94	2.05	0.15
2004	AB	C	4.00	0.08	17.2	13.7	1.15	0.93	79.6	13.9	3.86	1.82	0.67	0.11
2004	AB	DL	4.15	0.18	26.6	14.7	10.5	6.99	48.2	8.41	2.52	0.78	0.83	0.09
2004	AB	L	4.11	0.06	38.4	2.08	3.10	0.48	56.8	2.42	3.07	1.51	0.79	0.18
2004	AB	SF	4.29	0.07	30.8	17.0	0.95	0.86	67.2	18.6	4.25	0.93	0.72	0.09

Y	H	T	pH		CaECEC		MgECEC		AlECEC		P		SOM	
			Average	SD	Average	SD	Average	SD	Average	SD	Average	SD	Average	SD
2005	Ap1	C	3.74	0.06	3.40	2.10	0.51	0.28	86.9	1.36	17.0	1.50	2.33	0.08
2005	Ap1	DL	6.42	0.23	73.0	4.52	15.6	6.85	5.38	1.91	15.7	4.03	2.37	0.76
2005	Ap1	L	5.26	0.24	85.3	1.49	1.88	0.69	9.38	3.92	17.9	0.48	2.51	0.41
2005	Ap1	SF	6.50	0.68	88.0	5.67	0.48	0.20	10.2	5.69	15.5	1.03	2.21	0.13
2005	Ap2	C	3.76	0.04	8.30	7.10	1.25	0.61	86.6	8.95	20.1	2.22	2.30	0.23
2005	Ap2	DL	4.34	0.24	42.4	20.7	28.1	4.59	22.2	21.9	15.7	0.46	2.43	0.66
2005	Ap2	L	4.05	0.14	37.7	6.61	0.48	0.17	60.5	6.03	13.4	1.47	2.03	0.13
2005	Ap2	SF	4.18	0.29	50.7	23.2	0.25	0.21	48.2	22.8	14.1	1.05	1.89	0.28
2005	AB	C	3.84	0.03	9.90	7.46	0.73	0.49	87.8	9.53	12.4	7.05	0.65	0.22
2005	AB	DL	3.93	0.19	11.9	4.77	14.5	3.40	68.4	12.4	10.9	7.51	1.03	0.50
2005	AB	L	3.88	0.04	19.6	7.94	1.20	1.30	78.4	9.69	2.13	0.71	0.81	0.15
2005	AB	SF	4.04	0.18	27.4	19.0	1.29	0.13	69.6	20.6	13.2	8.88	0.85	0.25
2006	Ap1	C	3.93	0.09	9.45	6.19	1.30	1.40	82.1	7.64	16.3	0.96	2.54	0.44
2006	Ap1	DL	5.77	1.23	77.9	6.20	10.2	2.26	6.16	1.49	14.4	4.46	2.24	0.42
2006	Ap1	L	5.49	0.35	90.6	6.36	0.83	0.38	6.55	4.96	7.66	1.31	2.15	0.47
2006	Ap1	SF	6.48	0.80	92.2	1.78	0.53	0.15	5.88	0.73	10.2	1.66	2.28	0.18
2006	Ap2	C	4.07	0.14	4.58	1.17	0.98	0.13	88.5	0.72	11.5	0.75	1.72	0.38
2006	Ap2	DL	5.06	0.86	52.4	22.0	26.2	5.73	16.4	17.0	9.45	2.17	1.67	0.25
2006	Ap2	L	4.15	0.06	48.0	13.9	0.34	0.20	50.9	14.4	11.4	1.00	2.23	0.26
2006	Ap2	SF	4.34	0.01	74.7	7.47	0.61	0.12	23.7	7.35	12.5	5.00	1.90	0.14
2006	AB	C	3.92	0.06	18.3	8.47	2.09	0.40	79.0	9.56	7.90	5.77	0.48	0.04
2006	AB	DL	3.95	0.08	19.5	6.04	28.0	7.51	51.7	8.70	7.60	4.22	0.57	0.10
2006	AB	L	4.00	0.12	22.8	11.5	0.68	0.38	75.4	12.8	5.01	3.58	1.02	0.28
2006	AB	SF	4.10	0.12	23.4	2.59	1.10	0.12	72.7	7.54	6.49	5.84	0.58	0.24
2007	Ap1	C	3.92	0.06	8.75	4.81	2.38	0.46	86.1	6.24	14.6	2.52	2.22	0.31
2007	Ap1	DL	6.04	0.66	69.1	5.18	15.3	3.36	11.1	3.89	9.24	2.48	2.43	0.65
2007	Ap1	L	4.67	0.27	73.3	18.3	1.00	0.66	24.4	18.6	10.6	0.56	2.58	0.32
2007	Ap1	SF	6.29	0.20	79.1	2.91	2.18	0.24	14.9	7.41	10.4	0.74	1.98	0.14
2007	Ap2	C	3.98	0.05	4.35	3.42	2.64	0.54	90.7	2.24	12.3	0.67	1.92	0.46
2007	Ap2	DL	4.57	0.07	31.8	12.8	26.1	8.72	34.2	22.4	10.7	2.25	1.97	0.27
2007	Ap2	L	4.30	0.17	54.0	21.1	0.69	0.47	43.9	21.5	10.5	3.64	2.18	0.31
2007	Ap2	SF	5.17	0.28	54.4	3.91	2.55	1.81	35.3	9.12	9.74	3.57	2.19	0.35
2007	AB	C	4.05	0.06	6.28	5.63	2.55	0.54	89.1	6.01	9.35	6.92	0.58	0.18
2007	AB	DL	4.00	0.16	8.08	6.66	8.83	6.73	82.3	6.62	14.2	4.56	0.63	0.09
2007	AB	L	4.00	0.05	15.3	5.34	1.95	0.98	79.2	9.26	12.4	5.38	0.66	0.12
2007	AB	SF	4.15	0.12	6.34	5.43	1.71	0.54	90.0	5.67	16.4	3.60	0.68	0.21
2008	Ap1	C	4.07	0.11	7.50	7.58	2.45	1.34	84.0	4.24	16.0	3.48	2.12	0.15
2008	Ap1	DL	6.07	0.38	65.6	7.98	14.8	5.72	17.2	9.62	11.8	4.25	2.13	0.13
2008	Ap1	L	5.48	0.36	78.5	13.5	1.78	0.17	17.1	13.1	7.01	0.65	2.29	0.13

Y	H	T	pH		CaECEC		MgECEC		AlECEC		P		SOM	
			Average	SD	Average	SD	Average	SD	Average	SD	Average	SD	Average	SD
2008	Ap1	SF	5.67	0.27	88.7	1.63	0.68	0.24	10.3	1.39	14.4	4.58	2.20	0.17
2008	Ap2	C	4.11	0.04	7.83	7.25	2.33	0.82	84.7	6.85	15.5	3.35	2.07	0.48
2008	Ap2	DL	5.56	0.75	67.8	6.36	19.1	1.95	12.1	3.08	12.3	3.91	1.99	0.39
2008	Ap2	L	4.72	0.25	72.0	16.4	1.20	0.70	19.2	7.37	14.7	3.54	2.31	0.29
2008	Ap2	SF	5.16	0.49	66.8	15.8	1.18	0.28	31.6	16.2	15.8	6.43	2.28	0.36
2008	AB	C	4.16	0.15	9.50	5.54	1.09	0.70	86.6	5.69	12.1	4.39	0.89	0.14
2008	AB	DL	4.98	0.27	46.3	17.7	19.7	1.97	24.1	8.52	10.3	2.42	1.44	0.66
2008	AB	L	4.42	0.23	51.4	27.3	0.85	0.71	46.6	29.0	7.25	1.18	1.67	0.14
2008	AB	SF	4.66	0.43	40.4	8.74	0.88	0.82	52.1	3.72	11.7	3.09	1.34	0.36
2010	Ap1	C	3.83	0.09	5.13	2.33	1.95	1.22	88.1	3.11	20.6	0.95	2.03	0.41
2010	Ap1	DL	5.62	0.02	73.3	8.89	20.9	9.71	4.05	1.62	13.8	1.09	1.85	0.03
2010	Ap1	L	4.81	0.36	86.9	0.59	1.01	0.58	8.45	3.33	14.4	1.47	1.90	0.03
2010	Ap1	SF	5.65	0.16	92.9	2.93	0.89	0.14	4.70	2.60	13.1	2.99	2.05	0.11
2010	Ap2	C	4.12	0.38	6.51	3.09	3.40	1.88	81.7	0.78	8.94	1.18	1.77	0.31
2010	Ap2	DL	4.70	0.57	60.1	8.26	20.3	5.90	21.8	13.0	8.01	5.27	1.58	0.13
2010	Ap2	L	4.42	0.31	76.0	8.17	2.23	1.23	17.8	7.68	9.64	3.38	1.76	0.09
2010	Ap2	SF	4.69	0.55	90.9	2.56	1.61	0.25	5.59	2.10	8.45	2.06	1.81	0.11
2010	AB	C	4.31	0.35	6.08	6.70	1.69	1.00	87.9	5.09	3.65	0.79	0.70	0.13
2010	AB	DL	4.08	0.09	16.3	9.27	7.87	1.81	71.4	11.2	4.33	2.94	0.60	0.09
2010	AB	L	4.04	0.12	30.0	14.3	0.96	0.74	65.8	16.5	2.78	0.87	0.63	0.16
2010	AB	SF	4.11	0.10	39.1	6.47	2.33	1.50	56.4	5.05	3.39	1.27	0.68	0.04

Table S2. Averages and standard deviations (SD) of biomass (Spike: spike rye biomass; Stem: stem rye biomass (all of them in kg/ha)) and stem phosphorus levels (P-Rye (%)) during 2002-2010. Y: Year of sampling; T: Liming treatment (C: control; DL: dolomitic limestone; L: limestone; SF: sugar foam).

Y	T	Spike		Stem		P-Rye	
		Average	SD	Average	SD	Average	SD
2002	C	1190	79.3	1550	41.6	0.03	0.01
2002	DL	2260	181	2970	340	0.02	0.01
2002	L	2030	148	2740	322	0.02	0.00
2002	SF	2480	88.4	3160	709	0.04	0.01
2003	C	836	62.4	1270	166	0.03	0.01
2003	DL	1430	258	2030	243	0.04	0.02
2003	L	1340	72.9	2090	76.0	0.04	0.00
2003	SF	1430	31.4	2060	124	0.03	0.01
2004	C	520	142	693	42.8	0.02	0.00
2004	DL	1310	110	1670	187	0.02	0.01
2004	L	1000	193	1350	217	0.02	0.01
2004	SF	1110	346	1390	520	0.03	0.01
2005	C	578	27.9	1000	159	0.02	0.01
2005	DL	1180	42.7	1640	203	0.02	0.01
2005	L	1210	36.4	1720	104	0.02	0.01
2005	SF	1310	94.4	2010	119	0.03	0.01
2006	C	1350	294	1500	58.6	0.01	0.00
2006	DL	1860	63.0	2110	45.4	0.02	0.00
2006	L	1990	85.1	2310	171	0.02	0.01
2006	SF	1930	81.8	2200	336	0.02	0.00
2007	C	80.0	35.5	93.4	39.3	0.03	0.01
2007	DL	430	96.7	565	121	0.05	0.00
2007	L	450	116	633	77.5	0.05	0.01
2007	SF	550	157	450	89.0	0.07	0.00
2008	C	970	151	1470	220	0.03	0.01
2008	DL	1000	488	1430	766	0.05	0.00
2008	L	1220	279	1690	403	0.03	0.00
2008	SF	888	165	1300	299	0.04	0.00
2010	C	1300	20.9	1720	58.2	0.02	0.00
2010	DL	1710	133	2140	186	0.05	0.01
2010	L	1610	188	1950	138	0.03	0.01
2010	SF	1580	90.7	2010	227	0.03	0.00
